# Metagenome Analysis of Surface Waters of the Shardara Reservoir, the Largest Artificial Reservoir in Southern Kazakhstan

**DOI:** 10.1128/MRA.00053-20

**Published:** 2020-03-12

**Authors:** Madina S. Alexyuk, Andrey P. Bogoyavlenskiy, Pavel G. Alexyuk, Yergali S. Moldakhanov, Vladimir E. Berezin

**Affiliations:** a Research and Production Center for Microbiology and Virology, Laboratory of Antiviral Protection, Almaty, Kazakhstan; Georgia Institute of Technology

## Abstract

Here, we present a metagenomic analysis of the microflora of the surface waters of the Shardara reservoir, the largest artificial reservoir in Southern Kazakhstan, created to meet irrigation and hydropower engineering needs. In this case, shotgun metagenomic sequencing of the microbial community DNA was used.

## ANNOUNCEMENT

The Shardara reservoir is the largest artificial reservoir in Southern Kazakhstan, created to meet irrigation and hydropower needs by regulating the runoff of the Syr Darya River. The Shardara reservoir is one of the largest fisheries in Kazakhstan; therefore, the study of the diversity of its microbiome, phytoplankton, and zooplankton is one of the significant tasks of ecological monitoring in the region (1, 2).

In our research, a metagenomic study of the microflora diversity of the Shardara reservoir near the hydroelectric power plant was carried out. In this case, shotgun metagenomic sequencing of microbial community DNA was used, which, in our opinion, gave a more detailed picture of not only the bacterial diversity but also the viral and eukaryotic diversity. Water samples were collected from the surface of the Shardara reservoir near the hydroelectric plant at 1-week intervals in May 2019 (collection point coordinates, 41.244722, 67.972222).

Each of the water samples (sample volume, 5 liters) was immediately filtered using a cellulose membrane with a diameter of 300 mm and pore size of 3 μm; then, the filtrate was concentrated to a volume of 500 ml using tangential flow filtration (Vivaflow 200, Sartorius, with a 200-cm^2^ polyethersulfone membrane). The samples were pooled and further concentrated by ultracentrifugation at a speed of 29,000 rpm for 120 min at 4°C (Avanti J30I ultracentrifuge, Beckman Coulter). The pellet was resuspended in a minimal volume of phosphate-buffered saline. Finally, nucleic acids were extracted using a Pure Link genomic DNA minikit (Invitrogen) according to the manufacturer’s protocol. DNA libraries were prepared from 1 ng of the isolated nucleic acids using the Nextera XT DNA sample preparation kit (Illumina, USA) in accordance with the instructions. High-throughput sequencing was performed by using an Illumina MiSeq instrument (paired-end sequencing, 2 × 300 bp; MiSeq kit v3).

The obtained sequences were tested for quality using Trimmomatic (3) from the Genome Detective tool (4). After the removal of low-quality reads and adapter trimming (4), 2,169,637 reads were analyzed by the Kaiju program using the nonredundant protein database of bacteria, archaea, viruses, fungi, and microbial eukaryotes (NCBI BLAST *nr* +euk) with default parameters as a reference (5).

**FIG 1:**
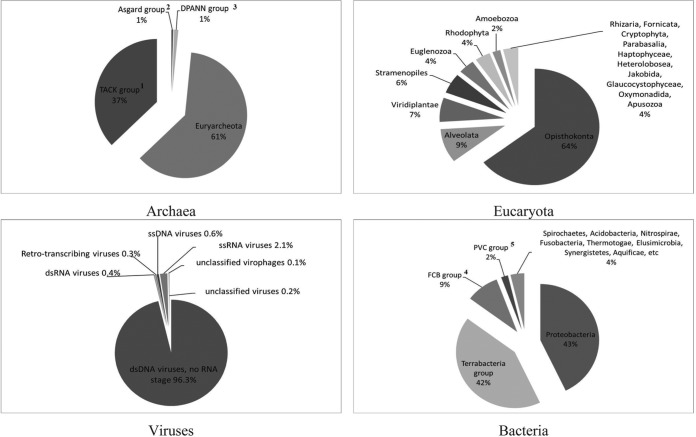
Taxonomic diversity of the microbial community of the Shardara reservoir. 1, The TACK group is a superphylum of *Archaea* that includes *Thaumarchaeota* and *Crenarchaeota*; 2, the Asgard group is a superphylum of *Archaea* that includes *Lokiarchaeota*, *Thorarchaeota*, *Odinarchaeota*, and *Heimdallarchaeota*; 3, the DPANN group is a superphylum of *Archaea* that includes “*Candidatus* Woesearchaeota,” “*Candidatus* Parvarchaeota,” and “*Candidatus* Micrarchaeota”; 4, the FCB group is a group of bacteria that includes *Fibrobacteres*, *Chlorobi*, and *Bacteroidetes*; 5, the PVC group is a group of bacteria that includes *Planctomycetes*, *Verrucomicrobia*, *Chlamydiae*, and *Lentisphaerae*. Taxonomic assignment of the metagenomic reads was based on the NCBI BLAST *nr* +euk database; Microsoft Excel 2016 was used to generate the plots.

Taxonomic classification of metagenomic data of the Shardara reservoir showed that 1% of the sequences corresponded to *Archaea*, 10% to lower eukaryotes, 3% to viruses, 82% to bacteria, and 4% to unclassified organisms (Fig. 1). This study is the first report on the bacterial and viral diversity of the Shardara reservoir based on a metagenomic analysis, which expands our knowledge of the microflora of the Aral–Syr Darya water basin.

### Data availability.

The raw sequence reads are available under BioProject accession number PRJNA601042.

## References

[1] Krupa EG, Barinova SS, Isbekov KB, Assylbekova SZ 2018 The use of zooplankton distribution maps for assessment of ecological status of the Shardara reservoir (Southern Kazakhstan). Ecohydrol Hydrobiol 18:52–65. doi:10.1016/j.ecohyd.2017.10.001.

[2] Krupa E, Barinova S, Assylbekova S, Isbekov K 2018 Structural indicators of zooplankton in the Shardara reservoir (Kazakhstan) and the main influencing factors. Turk J Fish Aquat Sci 18:659–669. doi:10.4194/1303-2712-v18_5_02.

[3] Bolger AM, Lohse M, Usadel B 2014 Trimmomatic: a flexible trimmer for Illumina sequence data. Bioinformatics 30:2114–2120. doi:10.1093/bioinformatics/btu170. 24695404 PMC4103590

[4] Vilsker M, Moosa Y, Nooij S, Fonseca V, Ghysens Y, Dumon K, Pauwels R, Alcantara LC, Vanden Eynden E, Vandamme AM, Deforche K, de Oliveira T 2019 Genome Detective: an automated system for virus identification from high-throughput sequencing data. Bioinformatics 35:871–873. doi:10.1093/bioinformatics/bty695. 30124794 PMC6524403

[5] Menzel P, Ng KL, Krogh A 2016 Fast and sensitive taxonomic classification for metagenomics with Kaiju. Nat Commun 7:11257. doi:10.1038/ncomms11257. 27071849 PMC4833860

